# From CD16a Biology to Antibody-Dependent Cell-Mediated Cytotoxicity Improvement

**DOI:** 10.3389/fimmu.2022.913215

**Published:** 2022-06-03

**Authors:** Loïs Coënon, Martin Villalba

**Affiliations:** ^1^ Institute for Regenerative Medicine and Biotherapy (IRMB), Univ Montpellier, Institut national de la santé et de la recherche médicale (INSERM), Montpellier, France; ^2^ Institut du Cancer Avignon-Provence Sainte Catherine, Avignon, France; ^3^ Institute for Regenerative Medicine and Biotherapy, Univ Montpellier, Institut national de la santé et de la recherche médicale (INSERM), Centre national de la recherche scientifique (CNRS), Centre hospitalier universitaire (CHU) Montpellier, Montpellier, France

**Keywords:** CD16a, NK cells, ADCC, monoclonal antibodies, cell therapy

## Abstract

Antibody-dependent cell-mediated cytotoxicity (ADCC) is a potent cytotoxic mechanism that is mainly mediated in humans by natural killer (NK) cells. ADCC mediates the clinical benefit of several widely used cytolytic monoclonal antibodies (mAbs), and increasing its efficacy would improve cancer immunotherapy. CD16a is a receptor for the Fc portion of IgGs and is responsible to trigger NK cell-mediated ADCC. The knowledge of the mechanism of action of CD16a gave rise to several strategies to improve ADCC, by working on either the mAbs or the NK cell. In this review, we give an overview of CD16a biology and describe the latest strategies employed to improve antibody-dependent NK cell cytotoxicity.

## Introduction

Immunoglobulins G (IgG) are essential for the immune system, and their characteristics have been harnessed to develop new innovative therapies, particularly in cancers and autoimmune diseases (1). They are composed of 4 chains, 2 heavy and 2 light, forming a Y-shape structure with distinct functions. The (Fab′)_2_ part is responsible for the specificity of the antibody to its target, and the second moiety, the fragment crystallizable (Fc), could be seen as a communication platform with the immune system. IgG and immune cell interaction take place through a family of receptors: the Fc receptors (FcR). In humans, the FcR family for IgG (FcγR) is composed of 6 receptors: FcγRI/CD64, FcγRIIa/CD32a, FcγRIIb/CD32b, FcγRIIc/CD32c, FcγRIIIa/CD16a, and FcγRIIIb/CD16b. Only CD64 is defined as a high-affinity receptor, while CD32b is the only inhibitory receptor (2, 3).

In humans, 4 subclasses of IgGs exist, IgG1, IgG2, IgG3, and IgG4. The different FcγRs bind with variable affinities to those subclasses (4). CD16a can interact with all of them, although IgG1 and IgG3 show the highest affinity (4).

Natural killer (NK) cells are innate lymphocytes that are very efficient in destroying stressed cells, such as virally infected or tumor-transformed cells (5, 6). Human NK cells mainly express CD16a, while CD16b is restricted to neutrophils (7). Of note, a subset of NK cells has been reported to express all CD32 variants (8). Human NK cells are divided into two main subsets: CD56^bright^ and CD56^dim^. CD56^bright^ NK cells are competent cytokine secretors but lack CD16a (9). CD56^dim^ are highly cytotoxic and express CD16a (10, 11). Upon recognition of IgG-opsonized targets through CD16a, NK cells trigger a potent cytotoxic mechanism called antibody-dependent cell-mediated cytotoxicity (ADCC), leading to the death of the target cell. This mechanism relies on the formation of an immunological synapse and the degranulation of lytic granules containing perforin and granzymes (12). Besides degranulation, NK cells can also eliminate target cells by engaging target death receptors, e.g., DR4, DR5, or Fas, with their death receptors ligands, e.g., FasL and TRAIL (13).

Cellular therapies based on T lymphocytes, more precisely on Chimeric Antigen Receptor (CAR) T cells, recently became an important weapon in the anticancer arsenal, with good efficiency in hematological cancers. However, achieving success in solid cancers is more challenging (14). Moreover, CAR T-cell therapy could induce very serious adverse events, such as graft-versus-host disease (GvHD), neurotoxicity, or cytokine release syndrome (15). Interestingly, NK cells do not induce them (16). Nevertheless, there are still some limitations to their large-scale use in clinics (17), and hence, there is a need to improve their clinical efficiency, in particular on ADCC to release the full clinical capacity of monoclonal antibodies (mAbs).

Here, we first review the basic knowledge of CD16a, and second, we show how this fundamental knowledge helps increase ADCC activity and present promising advancements for improving immunotherapy.

## CD16a Biology

### Genetic

CD16a is encoded by *FCGR3A* gene, which is located on the long arm of chromosome 1, in the 1q23 region. Two functional polymorphisms of the CD16a have been described, 158 V/F (18) and 48L/H/R (19), sometimes called 176 V/F and L66/H/R, respectively, depending on when counted from the N-terminal of the mature protein or not. The first polymorphism is due to a nucleotide substitution T to G at position 559. The second is due to two possible different substitutions at position 230, either T to G or T to A. The 48L genotype was first described to have a lower binding to IgG than the 48R or 48H genotype (19). Later, it was shown that the differences are due to the 158 V/F genotype rather than the L48/H/R genotype (20, 21). However, there is a link between 158V/F and 48L/H/R genotypes, with 48L being preferentially expressed together with 158F and 48H or 48R with 158V (22). CD16a-bearing 158V phenotype shows a superior binding to IgG (20, 21). This increase in affinity has at least two consequences. First, CD16a 158V shows a better ADCC *in vitro* (23) and gives a better clinical response to rituximab therapy, most likely through ADCC (21, 24). Second, V/V patients display a faster clearance of rituximab during their treatment, possibly due to the better recognition by CD16a 158V (25).

It was believed that the 158V/F polymorphism could impact the amount of CD16a expressed by NK cells, and being 158V, the one expressing more CD16a (26). However, a subsequent study showed that depending on the clone of anti-CD16 used on cytometry, certain differences can arise (27). In conclusion, the amount of CD16a expressed by NK cells is probably not related to the 158V/F genotype.

### Structure

CD16a is a transmembrane receptor with a short C-ter cytoplasmic tail and possesses two extracellular Ig-like domains (28). It does not possess any signaling component in its intracellular part. Thus, to transduce signals, it needs two immunoreceptor tyrosine-based activation motif (ITAM)-bearing signaling chains, as described later.

### CD16a/IgG Interaction

CD16a interacts in a 1:1 stoichiometry fashion with the lower hinge/upper CH2 of IgG. The Fc N-glycan chain, linked to the N297 of the CH2, also plays a critical role in this interaction (29, 30). Thus, not only the amino acid (31) but also the glycan composition (32) can greatly influence the affinity of CD16a for the Fc and consequently the potency of ADCC.

Besides IgG Fc interaction with CD16a, recent data support that the Fab could be implicated in the IgG/CD16a interaction (33, 34). Moreover, mutations in the Fab seem to modulate ADCC, highlighting the potential interaction of Fab-CD16a during IgG-CD16a binding (35).

### Glycosylation of CD16a

The Fc N297-linked glycan has a critical role in its interaction with CD16a (32). Less is known about the role of CD16a glycosylation and how it could impact the affinity for IgG. CD16a is decorated with 5 asparagine(N)-linked glycan at N38, N45, N74, N162, and N169 (7). The N-glycan composition of CD16a has been only recently solved, due to the difficulty to have enough material. Among the 3 donors studied, the N-glycans are composed of 23% of the high-mannose structure, 22% of hybrid type structure, and the remaining (55%) being complex type N-glycans (36). However, only the N162 N-glycan seems to directly impact CD16a affinity toward IgG, conversely with its position in the IgG-binding site (29, 30). Although the N45 N-glycan is not situated in the IgG-binding site, it also contributes to the binding to IgG (37) by stabilizing the CD16a structure (38, 39).

Finally, CD16a 48L/H polymorphism, which is close to the N45 glycan site, modulates the composition of its glycosylation (40) and potentially CD16a function. With the 158F/V polymorphism being also close to the N162 glycan, it is tempting to postulate that the same kind of relationship between amino acid sequence and N-glycosylation pattern could exist.

### Signaling Pathway

NK cell cytotoxicity is regulated by a plethora of inhibitory or activating receptors. When NK cells interact with a potential target cell, they receive activating and inhibiting signals through those receptors. If the balance is in favor of inhibition, the target cell will survive; if not, the cell will be killed (41). It is worth noting that some inhibitory signals are very strong and need a large involvement of activating receptors to be overcome (42). CD16a probably provides the strongest signals and can overcome the inhibitory signals. The engaged CD16a forms clusters in lipid rafts (43), a cholesterol-rich lipidic microdomain structure, and hence, CD16a signaling takes place in them (44), enabling proper intracellular signal (45, 46).

As described previously, CD16a does not possess any ITAM domain in its cytoplasmic tail and thus needs the help of a tandem of two intracellular chains bearing ITAM domains, CD3ζ and FcϵRIγ (47–49), which are indistinctly used (50). Noteworthy, the two signaling chains are covalently associated between them, but not to CD16a (51). A unique feature of the intracytoplasmic tail of CD16a, compared to other FcγR, is the possibility to be phosphorylated by PKC (52). The downstream signaling pathway of phosphorylated CD16a favors cytokine production, while the unphosphorylated leads to stronger degranulation (52).

Upon CD16a engagement, a kinase belonging to the Src family, Lck, becomes activated (53) and phosphorylates the ITAM domains of CD3ζ and/or FcϵRIγ (54). The phosphorylated ITAMs allow the recruitment and the phosphorylation of kinases from the Syk family (55), such as Syk (56) and ZAP-70 (54), which are in turn responsible for the subsequent signaling. Among their substrates, PI3K is highly relevant (57, 58), because it converts PIP2 to PIP3, which is processed by PLC-γ (55) releasing IP3 and DAG (59). DAG activates the PKC family, which contributes to the triggering of degranulation. IP3 induces calcium release from the endoplasmic reticulum to the cytosol (59). This calcium influx is one of the major signals for ADCC triggering and also allows NFAT translocation in the nucleus, inducing transcription of its target genes (60). There are other pathways activated by CD16a engagement that contribute to ADCC, e.g., the ERK2 MAPK pathway.

### Downregulation

After CD16a-dependent NK cell cytotoxicity, CD16a is rapidly downregulated. One of the mechanisms involved in the shedding of CD16a is mediated by a disintegrin and metalloprotease 17 (ADAM17) (61), which is expressed on NK cells. This metalloprotease cleaves the stalk region of CD16a between Ala^195^ and Val^196^ (62). The cleaving of CD16a by ADAM17 occurs in *cis*. This means that an ADAM17 expressing NK cell cannot induce CD16a shedding on another NK cell (62). Another mechanism of CD16a downregulation after activation is internalization, which occurs not only to CD16a but also to other intracellular signaling components such as CD3ζ, ZAP-70, and Syk. They are ubiquitinated, probably leading to their degradation (46, 62–64).

## Improving Natural Killer Cell Antibody-Dependent Cell-Mediated Cytotoxicity

**Figure 1:**
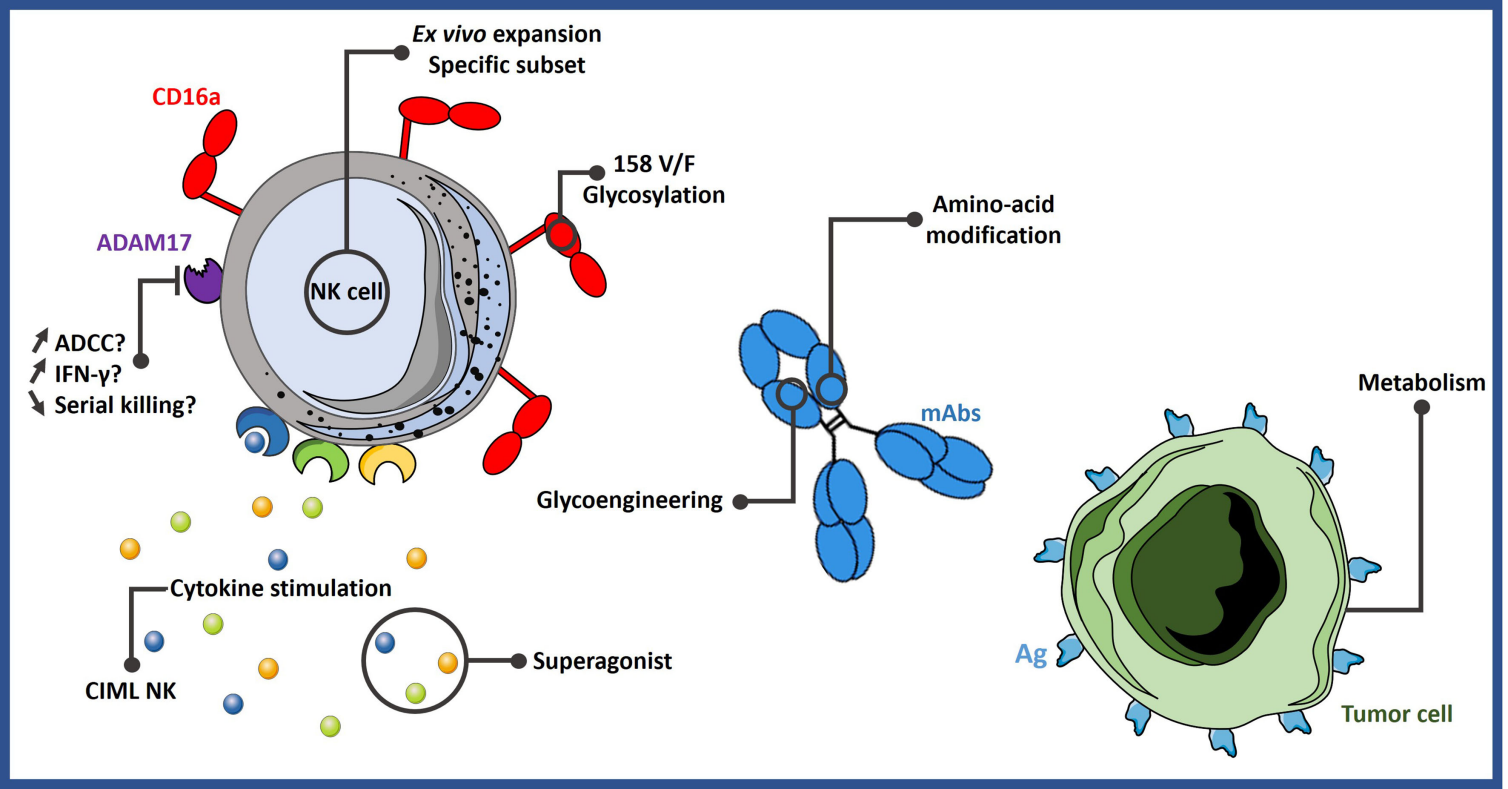
Different strategies to improve ADCC. NK cell-mediated ADCC can be enhanced by expanding them with specific protocols or by stimulating them with certain cytokine cocktails. It could be possible to select more potent NK cell subsets based on their phenotype or genotype. Engineering mAbs are also an efficient way to increase ADCC activity. Lastly, tumor cells can be sensitized to NK cell activity by modifying their metabolism. ADCC, antibody-dependent cell-mediated cytotoxicity; Ag, antigen; CIML NK, memory-like natural killer cells; mAbs, monoclonal antibodies.

The strategies described in this section are summarized in **Figure 1**.

### Making Highly Active Natural Killer Cells for Therapy

NK cell engraftment has been successfully performed in several clinical assays, for example, with the infusion of cytokine-induced memory-like (CIML) NK cells (65, 66). The most utilized cocktail contains IL-12, IL-15, and IL-18 (67, 68). CIML NK cells express more IFN-γ than conventional NK and show superior cytotoxicity against leukemia cells and primary acute myeloid leukemia (AML) blasts (65). Moreover, haploidentical CIML NK transfused in relapsed or refractory AML patients exhibited anti-leukemia function (65). CIML NK transfusion for relapsed pediatric AML patients post-hematopoietic cell transplant achieved complete remission in 4 out of 8 patients without significant toxicities (66), highlighting the potential of this strategy to treat leukemia. However, a strong effort is currently done to further expand these cells *ex vivo* before infusion in allogeneic settings. Since NK cell expansion has recently been reviewed (69, 70), we shortly comment on it. Primary NK cells can be expanded from peripheral blood or umbilical cord blood, usually using feeder cells. The culture media is usually supplemented with various cytokines, including IL-15 and IL-2, and accessory cells. NK cells have also been generated from induced pluripotent stem cells (iPSCs). In general, the resulting expanded NK cells show high cytotoxic activities. For clinical purposes, i.e., to mediate ADCC, it is interesting to produce NK cells with high CD16a expression, such as the protocol that we have recently described (71), which produces cells with efficient ADCC against primary tumor cells (72, 73).

Recently a new subset of NK cells has been described in healthy individuals with prior exposure to human cytomegalovirus (HCMV). HCMV infection induces a peculiar subset of NK cells, which are mainly characterized by the absence of the FcϵRIγ (74–76), one of the two chains responsible for CD16a signaling, as described before. Although these cells express a lower amount of natural cytotoxic receptors and thus show weak responsiveness toward the K562 tumor cell line compared to FcϵRIγ+ NK cells (74), they display an increased activity through Ab-dependent stimulation (74) and ADCC (75). Recently, a method to *ex vivo* expand these so-called “g-NK” cells has been published (77). Although the expansion is only possible from previously HCMV-infected donors, the resulting cells possess some interesting features. Consistent with previous research, g-NK cells induce stronger ADCC than conventional NK cells. Also, whereas NK cells express relatively high CD38 levels, g-NK cells have the particularity to express low or no CD38. This ectoenzyme is the target for the clinical mAb daratumumab (DARZALEX) used in multiple myeloma treatment (78). Although daratumumab shows benefit in the clinic, the patient’s NK cell count rapidly decreases during treatment (79). Thus, the uncommon g-NK cells phenotype is an advantage for their use in these patients. Taken together, g-NK cells could be a powerful anticancer agent when utilized in combination with cytolytic mAbs.

### Cytokine Priming

A simple way to improve ADCC activity is to stimulate NK cells with pro-inflammatory cytokines. IL-18 improves CD16a-induced IFN-γ production by NK cells after incubation on IgG-coated dishes. Moreover, IL-18-treated NK cells show increased ADCC with rituximab against the CD20+ Raji cell line, *in vitro* and *in vivo* (80).

IL-12 also increases IFN-γ and TNF-α production by NK cells after encountering trastuzumab-opsonized SKBR3 cells. Interestingly, a set of genes uniquely regulated by this co-stimulation have been identified. Among them, granzyme B was upregulated 10 times (81). IL-12 increases CD25 expression on NK cells (82). CD25 is the alpha subunit of the IL-2 receptor (IL-2Rα), which is composed of 3 subunits. The CD25/IL-2Rα is the subunit with the highest affinity for IL-2. The expression of CD25 by IL-12+ immobilized IgG-stimulated NK cells made them more sensitive to IL-2, particularly at low doses. In a phase I/II clinical trial, cetuximab was used along with IL-12 on patients suffering from unresectable or recurrent head and neck squamous cell carcinoma (83). Patients having experienced progression-free survival (PFS) longer than 100 days had their NK cells exerting more ADCC *ex vivo* as compared to patients with PFS shorter than 100 days. Thus, IL-12 priming of NK cells seems to increase their activity, particularly ADCC, even in treated patients. Remarkably, NK cells from these patients showed increased CD25 expression.

IL-15 is a cytokine essential for the survival and function of NK and CD8+ T cells (84). The IL-15 receptor is composed of 3 subunits, IL-15Rα (CD215), IL-15Rβ (CD122), and IL-15Rγ (CD132). The IL-15Rα is a high-affinity receptor for IL-15, although it is not expressed by NK cells but rather by antigen-presenting cells (APCs), such as monocytes and dendritic cells. APCs use the IL-15Rα to transpresent IL-15 to NK cells expressing the two other subunits (85). An IL-15 superagonist was described using this IL-15Rα subunit (86). This fusion protein between IL-15 and IL-15Rα displays stronger activity than the native IL-15 (86). Both native IL-15 and the IL-15/IL-15Rα construct have reached clinical trials. A 5-day IL-15 intravenous administration to various solid cancer patients leads to a 34-fold increase in NK cell counts (87). Treatment also increased NK cell-mediated ADCC *ex vivo*. This short-term IL-15 administration was well tolerated. Other recent clinical trials are using the fusion protein IL-15/IL-15Rα, such as NIZ985 in a phase I dose-escalating study as single-agent therapy in advanced solid cancers patients (88). Following treatment, NK cells and CD8+ T lymphocytes proliferated, and this was associated with increased plasma levels of IFN-γ, IL-18, and CXCL10. NKTR-255 is a polyethylene glycol-conjugated human IL-15, designed in an attempt to increase the pharmacokinetics (PK) of IL-15 while retaining the ability to interact with its receptor (89). In an *in vivo* model, NKTR-255 showed a better PK and an increase of granzyme B and CD107a positive NK cells compared to IL-15 or IL-15 complexed with IL-15Rα, as well as better survival in mice bearing Daudi lymphoma xenografts. NKTR-255 is currently investigated in a phase I trial as monotherapy or in combination with daratumumab or rituximab for the treatment of refractory multiple myeloma or non-Hodgkin’s lymphoma, respectively (NCT04136756). N-803, previously known as ALT-803, is a mutated N72D superagonist IL-15 bound to IL-15Rα and fused to an IgG1 Fc that showed enhanced NK cells activity *in vitro* and *in vivo* in preclinical models (90, 91). Recently, the PK and safety of N-803 have been assessed in healthy volunteers. It did not produce adverse events and persisted in circulation ~10-fold longer as compared to IL-15. Moreover, it increased NK and CD8 T-cell numbers (92). N-803 has been used instead of IL-2 to support cytokine-primed allogeneic NK cells transfer to AML patients in a relapse in two separate clinical trials (NCT03050216 and NCT01898793). Unexpectedly, the report stated that the use of N-803 instead of IL-2 led to reduced clinical activity (93). The authors showed that N-803 promotes recipient CD8+ T-cell activation, inducing a decrease in allogeneic NK cell persistence through rejection. Although IL-15-based immunotherapy could be seen as a promising strategy to support NK cell therapy, the best clinical protocol remains to be described.

### ADAM17 Inhibition: A Double-Edged Sword?

As described above, ADAM17 is the main driver of CD16a downregulation after activation. One strategy to improve NK cell ADCC is to prevent CD16a shedding by targeting ADAM17. Some conflicting reports are published. A patient with a rare genetic deficiency resulting in an absence of expression of ADAM17 provided some insights into the relevance of this strategy (94). The patient’s NK cells showed a strong defect in cytokine secretion, while the response to CD16a engagement was similar to that of 2 healthy donors, in terms of IFN-γ production as well as ADCC efficiency. However, blocking ADAM17 activity in NK cells from healthy donors, by either a chemical or an antibody, increases IFN-γ production after antibody stimulation (61, 95). Similarly, others published that ADAM17 knock-out using CRISPR/Cas9 technology in purified NK cells from peripheral blood mononuclear cell (PBMC) shows better IFN-γ production and ADCC activity *in vitro* and *in vivo* (96). It is possible that the constitutive deficiency of ADAM17 in that patient-generated NK cells does not depend on this enzyme. Nevertheless, ADAM17 blocking by chemicals has been shown to reduce the survival of the NK cells as well as the CD16a-mediated serial killing, the cells being unable to detach from the target cells, impeding their motility and preventing them to go to another target (97).

In conclusion, the benefit of ADAM17 inhibition is still unclear. The success of this strategy will be unveiled by a clinical trial in process (NCT04023071) where the use of iPSC-derived NK cells bearing non-cleavable CD16a (98) will be assessed in AML and B-cell lymphoma.

### Working on Monoclonal Antibodies

As a well-characterized protein, IgGs have been modified in their amino acid sequence to increase the Fc affinity to CD16a and, subsequently, improve ADCC (31, 99–101). Some mutations became “famous,” for example, the so-called GASDALIE, consisting of 4 substitutions in the Fc: G236A/S239D/A330L/I332E (102). This mutation shows a great increase of affinity to CD16a with almost no increase in CD32b affinity, which is the only inhibitory FcγR (103). Another mutation is referred to as Variant 18 (F243L/R292P/Y300L/V305I/P396L) and showed a remarkable increase in ADCC (104). These mutations have now reached the clinic as an anti-HER2 antibody, the margetuximab, and showed good results in phase I for the treatment of various HER2-positive carcinomas (105). Margetuximab is currently tested in breast cancer patients with chemotherapy versus trastuzumab plus chemotherapy (phase III SOPHIA trial). The preliminary results showed an increase in PFS for margetuximab compared to trastuzumab (106).

As stated previously, the N297 situated in the CH2 of the Fc region is linked to an N-glycan chain, which is extremely important for the interaction with all FcγRs, as aglycosylated IgGs cannot interact with them (107). Thus, glycoengineering the IgG Fc N-glycan holds great promise to increase affinity to CD16a (32). The most studied glycomodification is afucosylation, where the fucose attached to the first *N*-acetyl-glucosamine of the N-glycan chain is absent. This modification leads to an increased binding to CD16a and an improvement in ADCC (108). This has been used to generate the anti-CD20 antibody obinutuzumab (previously GA101), which displayed high efficacy *in vitro* and *in vivo* on monkeys (109). Moreover, afucosylation increases effector function through CD16a only if a certain amount of CD16a is expressed, such as on NK cells, in opposition with macrophages (110). It has also been described that afucosylated antibodies increase the IFN-γ secretion, as well as the serial killing (111). Finally, obinutuzumab reached the market under the brand name GAZYVARO in 2013 in the United States and in 2014 in Europe.

### Natural Killer Cell Engagers

Apart from changes in the amino acid sequence and glycoengineering of Fc IgG, it is also possible to work on the format of the antibodies. Based on the success of the bispecific T-cell engager (BiTE) strategy, a similar format was developed for NK cells, the so-called bispecific Killer cell engager (BiKE). This format consists of two single-chain fragment variables (scFv) linked between them through a linker. One scFv targets CD16a, while the other targets a tumor antigen (112). The trispecific Killer cell engager (TRiKE) format also exists, in which 2 tumor antigens are targeted along with CD16a, even if others add IL-15 in place of the third scFv (112, 113). The use of such a construct allows a retargeting of NK cells to the tumor cells and leads to a strong ADCC against target cells. The majority of BiKE/TRiKE are developed for hematological cancers, such as the CD16xCD33 BiKE, which shows efficient ADCC against samples from AML patients and can reactivate patient NK cells (114). AFM13 is a tetravalent bispecific antibody targeting CD16a and CD30, which has shown interesting results in a phase Ib trial in combination with pembrolizumab for treatment of refractory or relapsed Hodgkin lymphoma (115). Four other clinical trials are scheduled for AFM13 (115). GTB-3550 is a TRiKE CD16/CD33/IL-15 that is currently being studied in a phase I/II trial for several types of leukemia (NCT03214666).

Researchers and clinicians are also trying to develop NK cell engagers for solid tumors. A TRiKE CAM1615HER2, which contains a VHH targeting CD16a, an scFv for HER2 and IL-15, shows an increase in NK cell proliferation and activation *in vitro* and tumor clearance *in vivo* (116). AFM24 is a tetravalent bispecific antibody, composed of one full-length IgG with two scFv linked on the C-terminal of the CH3, which targets CD16a and EGFR. It showed better activity than cetuximab in ADCC, regardless of the mutational status of the cell line. In monkeys, AFM24 seems to be harmless (117). Taken together, the NK cell engager/retargeting strategy seems promising by increasing NK cell activity, in particular regarding ADCC.

### Increasing Natural Killer Cell Activity Through Metabolism: The Newest Field in Immunotherapy

It is known that metabolism dysregulation is a key driver of immunosuppression in the tumor microenvironment and also a cause of immunotherapy resistance (118). Many teams have shown that immune cell metabolism is critical for their function (119, 120). Recently, reports about phenotypic changes in leukemia cell lines through metabolic drugs show promising results regarding NK cell activity. Metformin, a drug originally used to treat type 1 diabetes, increases the expression of ligands, notably ICAM-1, which is recognized by lymphocyte function-associated antigen 1 (LFA-1). This is an integrin that also modulates lymphocyte intracellular signaling (121), including in NK cells (122, 123). Although metformin alone was not toxic for the cells, the combination with UCB-expanded NK cells (71) showed higher tumor cell clearance *in vitro* and *in vivo* (124). Another report shows that dichloroacetate (DCA), an inducer of oxidative phosphorylation (OXPHOS) metabolism, increases several stress ligands on leukemic cell lines, sensitizing them to NK natural cytotoxicity. However, this mechanism is linked to the p53 mutational status (125). Although these two reports highlight the potentiation of NK natural cytotoxicity on tumor cells by metabolic drugs, it is possible that this could increase as well ADCC. Further studies are needed to verify this.

Another strategy is to directly modulate NK cell metabolism. cMyc is a critical factor that regulates the metabolic machinery supporting glycolysis and OXPHOS in mouse NK cells. Lack of cMyc results in an impaired NK cell response (126). Glycogen synthase kinase-3 (GSK3) can mediate cMyc degradation in murine NK cells (126). Consistent with this, GSK3 overexpression has been detected in NK cells from AML patients, and this is linked to impaired cytotoxicity against AML cells. GSK3 inhibitors restore the NK cell activity in these AML patients against AML cell lines and primary AML cells. Moreover, expanded NK cells from donors treated with GSK3 inhibitors show superior activity in a mouse xenograft model of AML (127). Finally, NK cells treated with a GSK3 inhibitor during expansion resulted in enhanced tumor clearance in a xenograft mouse model of a human ovarian cancer cell line (128). As a promising approach, several clinical trials evaluated the efficacy of the GSK3 inhibitor LY2090314 in solid and hematological cancers (NCT01632306, NCT01287520, and NCT01214603). To our knowledge, the results are not published yet.

## Conclusion

Immunotherapy has shown exciting results in cancer patients, particularly for hematological malignancies. However, further progresses are still needed to achieve further success in solid cancer. Even being a long-last studied FcγR, the CD16a has still some features that need to be unraveled. With the advancement in technology, new findings and subsequent clinical approaches are expected. This knowledge will allow us to select the most suitable NK cells bearing the most efficient CD16a to exploit the full potential of clinical mAbs.

## Author Contributions

All authors were involved in preparing and writing the manuscript.

## Funding

This work was supported by INCA/DGOS PRT-K program 2021 (MV; 2021-014) and from the 2021 AAP Companies On Campus by the MUSE (Montpellier Université d’Excellence (MV). LC is a recipient of a fellowship from MRT. This work was also supported by the “Investissements d’avenir” Grant LabEx MAbImprove: ANR-10-LABX-53 (MV).

## Conflict of Interest

The authors declare that the research was conducted in the absence of any commercial or financial relationships that could be construed as a potential conflict of interest.

## Publisher’s Note

All claims expressed in this article are solely those of the authors and do not necessarily represent those of their affiliated organizations, or those of the publisher, the editors and the reviewers. Any product that may be evaluated in this article, or claim that may be made by its manufacturer, is not guaranteed or endorsed by the publisher.
